# Splenic angiosarcoma with hepatic and cardiac metastases: a case report and literature review

**DOI:** 10.3389/fonc.2025.1682054

**Published:** 2025-10-24

**Authors:** Fangzheng Chen, Ding Zhao, Haotian Yu, Shuai Wang, Yahao Wang, Haitao Lv

**Affiliations:** ^1^ Department of Hepatobiliary Surgery, Second Hospital of Hebei Medical University, Shijiazhuang, China; ^2^ Ophthalmology, Hebei Medical University Third Hospital, Shijiazhuang, China

**Keywords:** angiosarcoma, cardiac tumor, liver metastasis tumor, splenic tumor, splenic imaging

## Abstract

Splenic angiosarcoma (SA) is an rare malignant tumor in clinical practice, characterized by high malignancy, atypical early symptoms, and a dismal prognosis. In recent years, growing attention has been focused on its diagnosis and treatment. This report describes a patient who was admitted to the hospital due to left upper abdominal pain. Contrast-enhanced CT indicated unevenly enhanced tumors in the spleen, liver, and right atrium. The patient was treated with laparoscopic splenectomy + partial hepatectomy and was diagnosed based on postoperative pathology and immunohistochemistry (positive for CD31 and CD34). After the operation, anlotinib + toripalimab targeted immunotherapy was used, and the patient recovered well. This case suggests that SA is prone to metastasis in the early stage. Clinically, the diagnosis of SA requires a combination of pathological diagnosis and imaging diagnosis, and postoperative targeted immunotherapy may improve the prognosis.

## Introduction

SA is a malignant mesenchymal tumor that originates from the endothelial cells of the splenic sinusoids (1). It accounts for less than 2% of all sarcomas. It is a rare disease with an incidence rate of only 0.15 to 0.26 per million people (2). Since Langhans first reported the disease in 1879, there have been only about 300 related reports worldwide up to now, so SA remains a poorly understood entity.

SA is clinically rare and has an extremely high metastasis rate. Moreover, most patients are diagnosed with multiple metastases at the time of diagnosis. The incidence of SA is extremely low, and its early clinical symptoms are atypical. Patients usually present with atypical signs such as abdominal pain, fatigue, anemia, and thrombocytopenia (3). Therefore, SA is usually discovered at an advanced stage and has a poor prognosis.

The etiology of this disease remains unclear and serum-specific biomarkers are lacking. Diagnosis requires a combination of imaging examinations and histopathological examinations, supplemented by immunohistochemical detection of vascular differentiation markers (4).

In terms of treatment, splenectomy is the most important treatment method, but it rarely achieves a radical cure. Recent studies have shown that adjuvant chemotherapy has shown promise in improving survival, with median overall survival (OS) extending from 4 months to over 12 months in treated patients, even in those with large tumors (>5 cm) or metastases (5). In recent years, some studies have also explored the potential application of new treatment methods such as combined therapy with anti-PD-1 inhibitors and anti-VEGF tyrosine kinase inhibitors, which have achieved complete remission in individual cases, bringing new hope for the treatment of SA (6).

Herein, we report a SA case with concurrent liver and cardiac metastases—an extremely rare presentation. We discuss the diagnostic and therapeutic decision-making, and review relevant literature.

## Case description

**Figure 1:**
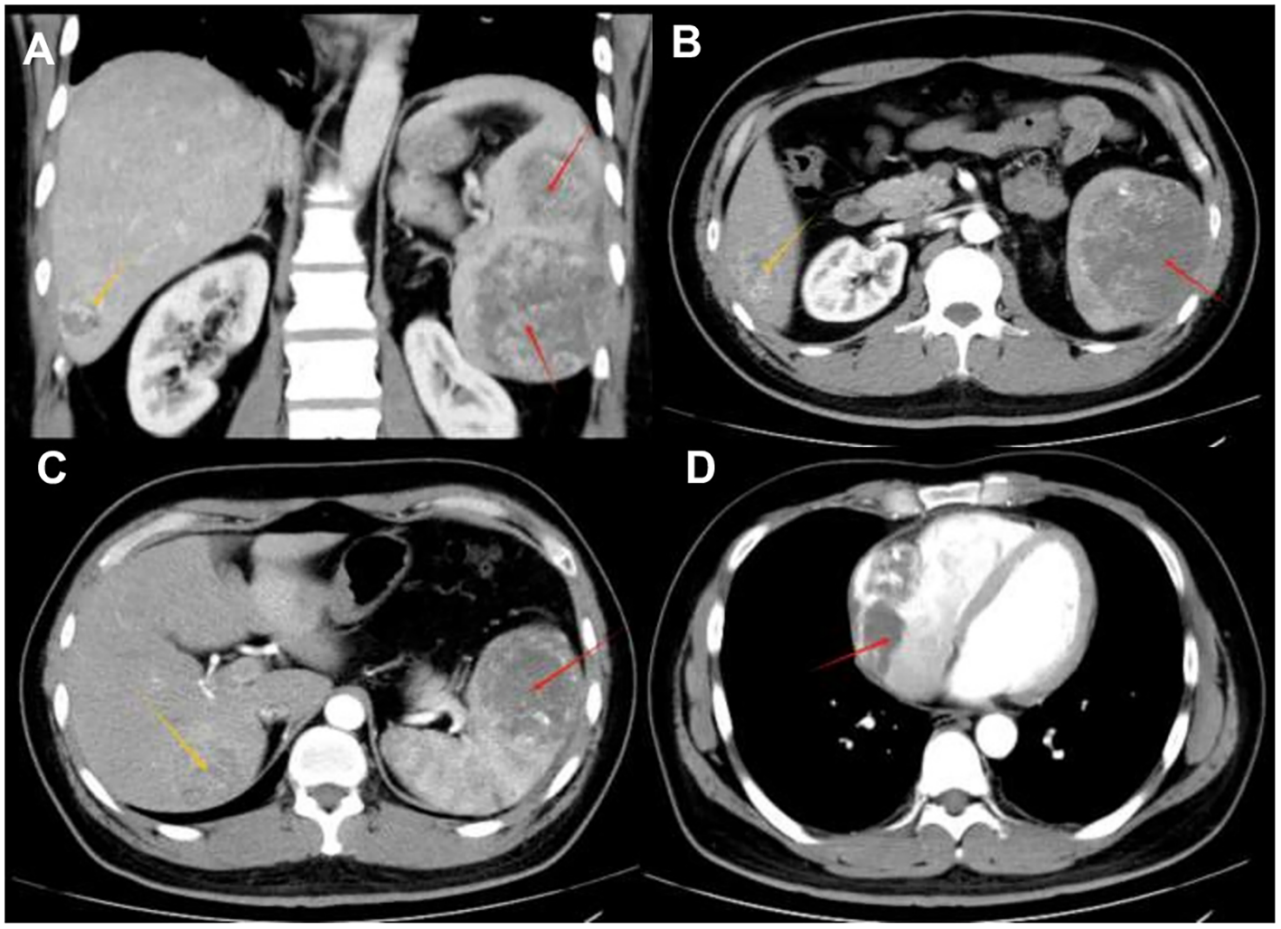
Radiological findings **(A)** A tumor within the spleen can be seen compressing the left kidney in the coronal position. **(B)** Contrast-enhanced CT shows multiple circular mixed-density lesions in the spleen. The size of the lesion in the inferior pole of splenic area is approximately 7.3 cm×6.3 cm×13.6 cm (indicated by the red arrow). Unevenly enhanced lesions can be seen in the V segment of the liver, approximately 3.7 cm×4.1 cm×4.5 cm in size (indicated by the yellow arrow) —consistent with typical SA liver metastasis. **(C)** An unevenly enhanced lesion with a size of approximately 4.1 cm×4.0 cm×3.5 cm (indicated by the yellow arrow) can be seen in segment VII of the liver. The lesion in the superior pole of the spleen is approximately 7.3cm×6.8cm×7.0cm in size (indicated by the red arrow). **(D)** Contrast-enhanced CT shows an unevenly enhanced lesion in the right atrium, with a maximum diameter of 7.1 cm (as indicated by the red arrow) —a rare metastatic site in SA, reported in only 1% of cases.

The patient is a 35-year-old male who was admitted to the hospital due to intermittent severe pain in the left abdomen for more than two months. Regarding the tumor, vascular computed tomography angiography (CTA) (**Figure 1**) revealed multiple circular mixed-density lesions in the spleen and the splenic tumor was compressing the left kidney. The tumor in the inferior pole protrudes locally outside the spleen contour, with a size of approximately 6.3 cm×7.3 cm×13.6 cm. Besides, CTA showed multiple circumscribed lesions with circular and heterogeneous enhancement in the liver. The larger one was located in segment VII of the liver, with a size of approximately 4.1 cm×4.0 cm×3.5 cm. CTA showed an inhomogeneous enhancement tumor in the right atrial region, with a maximum diameter of 7.1 cm. Due to the presence of surface ulcers and perisplenic hemorrhage in the patient’s splenic tumor, there is an extremely high risk of life-threatening spontaneous rupture. Additionally, the patient has asymptomatic cardiac metastasis without acute heart failure or hemodynamic instability. We performed laparoscopic partial hepatectomy and laparoscopic splenectomy on the patient.

Intraoperative observations: We found that the patient had a tumor of approximately 3 cm^3^×2 cm^3^×2 cm^3^visible in the V segment of the liver. In addition, a tumor could be seen near the lower edge of the patient’s spleen, with ulceration visible on the surface. Furthermore, a small amount of blood could be seen around the patient’s spleen. After we examined that there were no other metastases in the patient’s abdominal cavity, we removed the tumor in the V segment of the liver 2 cm along the tumor edge by using the forceps method. Then, we completely remove the spleen. The patient received regular dressing changes, and the patient recovered well.

**Figure 2:**
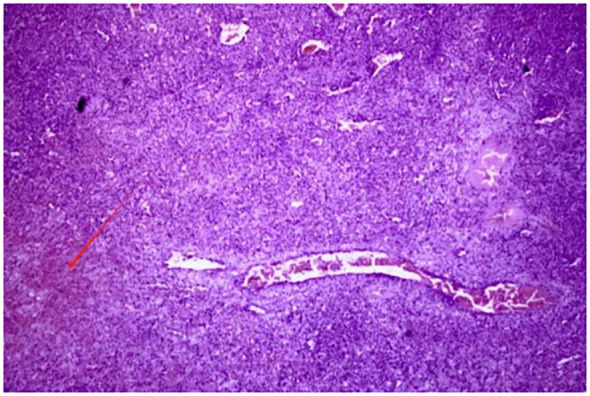
Postoperative pathological examination shows that the tumor was composed of atypical epithelioid cells. No cortical or medullary structures were seen in the spleen. A large number of proliferating tumor cells were observed in the field of view, and some showed necrosis (as indicated by the red arrow) —key histological features distinguishing SA from benign vascular tumors.

Postoperative pathology showed that the tumor was composed of atypical epithelioid cells, and no cortical or medullary structures were observed in the spleen. A large number of proliferating tumor cells and some cells showing necrosis (indicated by red arrows) could be seen in the field of vision, which was consistent with angiosarcoma (**Figure 2**). The liver metastatic lesions also conform to the characteristics of angiosarcoma, and the resection margins are negative. The immunohistochemistry results were as follows: Scavenger Receptor Cysteine-Rich Family Member 1(CD163) (scattered focal +), vascular endothelial marker (CD31) (+), (CD34) (+), CD68 (scattered +)、CD8 (scattered +), pancytokeratin (CKpan) (–), transmembrane glycoprotein (D2-40) (-), Epithelial Membrane Antigen (EMA) (-), ETS-Related Gene (ERG) (+), Factor VIII-related antigen (FVIII-Rag) (partial +), Human Herpesvirus 8(HHV-8) (-), Proliferating Cell Nuclear Antigen Ki-67(Ki-67) (approximately30% +), and smooth muscle actin (SMA) (-). More than one month after the operation, the patient visited the oncology department of our hospital and received targeted immunotherapy with anlotinib + toripalimab. Then the patient received treatment at another hospital, so we lost follow-up (**Table 1**).

**Table 1:** Patient treatment process.

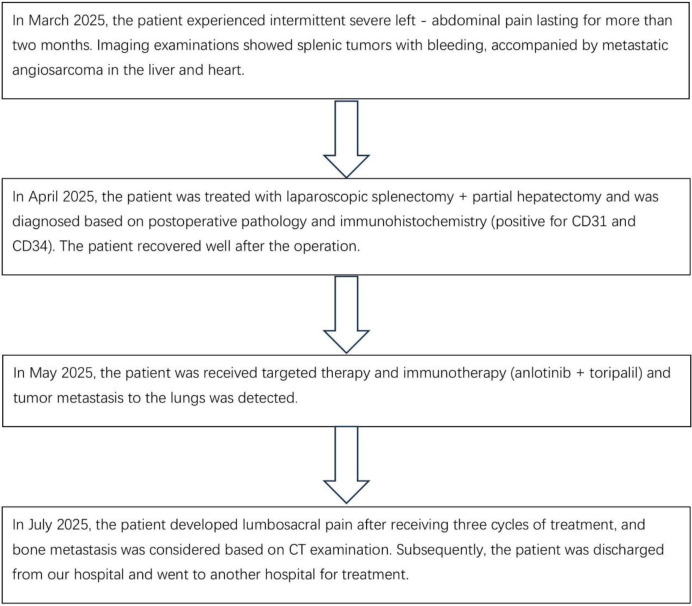

## Discussions

Case reports of SA highlight the complexity and aggressiveness of this rare malignancy. In the context of relevant literature, we summarized the initial symptoms, metastatic sites, therapy, and outcome and follow-up of this disease (**Table 2**), with the aim of providing valuable insights for clinicians in the diagnosis and management of this rare condition.

**Table 2:** Summary of representative literature on splenic angiosarcoma (2012 –2025).

Case	Author/Year	Age/sex	Initial symptom	Metastatic sites	Therapy	Outcome and follow-up
1 (6)	Xu W et al/2022	57/female	Abdominal pain	Liver	targeted immunotherapy	Alive, 8 months
2 (7)	Kamocki Z et al/2013	54/male	Abdominal pain and loss of appetite	Liver	Surgery	Death after 3 months
3 (7)	Kamocki Z et al/2013	77/female	Haematochezia and fatigue	Colorectal junction	Surgery	Death after about 1 month
4 (8)	Dirven I et al/2024	70/female	Abdominal pain	No	Surgery	Death after 4 years
5 (8)	Dirven I et al/2024	36/female	Abdominal pain	Liver	Chemotherapy	Lost to follow- up after a few months
6 (8)	Dirven I et al/2024	75/female	Fatigue and weight loss	Liver and spine	No	Death after 3 months
7 (10)	Ferreira BP et al/2014	57/female	abdominal pain, weight loss, decreased appetite and dyspnea	Liver	Surgery and chemotherapy	Alive, 2 years
8 (10)	Ferreira BP et al/2012	30/male	abdominal pain, night sweats, fevers, weight loss and fatigue	stomach	Surgery and chemotherapy	Death after 8 months
9 (11)	Myoteri D et al/2014	82/female	weight loss, fatigue, and mild afternoon fever	No	Surgery	Alive, 6 months
10 (12)	Chen X et al/2018	35/female	No	No	Surgery	Death after 4 years
11 (13)	Masuda T et al/2025	76/male	abdominal discomfort together with anemia and thrombocytopenia	Liver	Surgery and chemotherapy	Alive, 3 months
12 (17)	Yang KF et al/2016	51/female	abdominal pain and anorexia	Liver	Surgery	Death after 39 days
13 (20)	Teco-Cortes JA et al/2021	49/male	abdominal pain	Liver	Surgery and chemotherapy	lost follow-up
14 (21)	Batouli A et al/2016	45/female	abdominal pain	Liver and greater omentum	Surgery and chemotherapy	Death after 5 months

SA, as a malignant hemangioendothelioma of the spleen, is a highly malignant tumor derived from mesenchymal cells. SA differentiates from the splenic sinuses into endothelial cells and is extremely rare in clinical practice. At present, the etiology of SA in clinical research is not clear. Previous research report has found that SA was associated with a history of exposure to chemicals (vinyl chloride, thorium dioxide, and arsenic), radiation exposure, and benign splenic hemangioma (7). The main group affected by SA is men aged 50 to 70, and there is no genetic predisposition (8). Due to the abundance of blood sinuses in the spleen, SA is prone to spread through the bloodstream in the early stage, especially flowing back to the portal vein via the splenic vein. Therefore, approximately 70% of SA cases will develop liver metastasis. Cardiac metastasis is exceptionally rare: a review of 205 global SA cases (9) showed only 3 cases with cardiac involvement (1 in left ventricle, 2 in right atrium), and none with concurrent liver and right atrial metastases. The patient presented with metastatic tumors in both the liver (segments V/VII) and the right atrium, which expands the known metastatic spectrum of SA and highlights the unpredictable invasiveness of SA.

The average survival time of SA patients ranges from 4.4 months to 14 months, and only about 20% of patients can survive for more than 6 months (10). Therefore, the early diagnosis and treatment of SA are of great significance. However, SA patients usually lack specific clinical features. Most of them are admitted to the hospital with abdominal pain, accompanied by signs such as fatigue, splenomegaly, and weight loss. As the tumor progresses further, some patients may experience spontaneous splenic rupture, which can be life-threatening (11). The patient in this article was also admitted to the hospital for treatment due to spontaneous splenic rupture and bleeding. Decreased hemoglobin is the main manifestation of SA. In addition, some patients also show symptoms such as decreased white blood cells and platelets, and increased erythrocyte sedimentation rate (12). Previous report has indicated that thrombocytopenia in some patients was due to extensive liver metastasis of SA (Kasabach-Merritt phenomenon) (13). However, the manifestations such as pancytopenia caused by SA were very similar to those of hematological diseases. Moreover, the tumor markers of the majority of patients are within the normal range. Therefore, most SA patients are easily missed or misdiagnosed in clinical practice at the beginning.

Most SA patients do not have typical clinical symptoms and need to be differentiated from lymphoma, splenic metastatic tumors, benign splenic vascular tumors, splenic abscesses, and other splenic diseases Hence, imaging examinations (ultrasound, CT, and MRI) are of great significance in the diagnosis of SA. Ultrasound examination is the first choice for the initial examination of SA. Some patients showed solid heterogeneous tumors with anechoic spaces under ultrasound, which represented the characteristic vascular spaces of the tumors (14). However, most patients might only see mild splenomegaly in most cases. Therefore, ultrasound examination does not have specificity.

CT has high value in evaluating the general characteristics and complications of SA. Heterogeneous splenic tumors can be observed in 60% cases by CT. They presented as low-density shadows of varying sizes and densities, with irregular shapes and unclear boundaries from the surrounding lesions. Some lesions might fuse with each other (15). High attenuation areas shown by CT might indicate acute bleeding or hemosiderin deposition. Contrast-enhanced CT scans revealed significant differences between the enhanced and non-enhanced areas of the spleen immediately after the injection of contrast agent. The areas that have not been enhanced were mostly lesions with poor blood supply or ischemic necrosis. The lesions started to enhance after 10 min to 50 min, which was a sign of delayed enhancement of SA. These CT manifestations can be used to differentiate lymphoma from metastases. Nodules with enhanced margins could be seen in the lesion within the liver parenchyma, and necrosis in the center of the lesion presents as low-density shadows when liver metastasis occurs in the lesion (16).

In MRI scans, T1-weighted MR Images often show low signals in the center and periphery of the lesion, and focal high-signal areas can be seen in the necrotic center of some lesions. T2-weighted MR Images show that the lesions of the spleen and liver present as heterogeneous high-signal lesions with indistinct boundaries. These nodular lesions that show decreased or increased signal intensity are associated with necrosis, hemorrhage or fibrosis within the tumor, which can reflect the content of hemosiderin deposition and the degree of hemorrhage (17). Angiography was more specific in diagnosis. However, angiography is an invasive procedure and can cause certain harm to patients. Moreover, angiography cannot distinguish benign cavernous hemangioma lesions. Therefore, angiography is rarely used in clinical practice for SA examination.

Elhakim et al. (18)reported that endoscopic ultrasound (EUS, with a frequency of 5–10 MHz) has a higher spatial resolution (up to 0.1 mm), which can clearly display the heterogeneity, necrosis, and local infiltration of tumors. Contrast-enhanced endoscopic ultrasound (CE-EUS) using contrast agents such as SonoVue^®^ can evaluate tumor vascular distribution (e.g., rapid regression in malignant tumors) (19), which helps distinguish cardiac metastases of SA from benign cardiac lesions (such as myxomas). Integrating EUS into the multimodal imaging workflow for SA (CTA for initial staging + EUS for cardiac/peri-vascular details + pathological confirmation) can improve diagnostic accuracy and provide a basis for treatment strategies. This is consistent with recent recommendations that EUS should be considered for SA patients with suspected cardiac metastases (18, 19).

The diagnosis of SA in most patients relies on puncture biopsy or postoperative pathological diagnosis. However, spleen biopsy is usually contraindicated because puncture biopsy has a high risk of inducing spleen rupture and tumor metastasis, and the diagnosis rate is not high. Hence, SA can only be diagnosed through the gross morphology, histological morphology and specific histopathological examination of the tumor after splenectomy in most cases. It is visible to the naked eye that SA often shows signs of bleeding and necrosis. Besides, previous report has indicated that SA typically presented as diffuse tumors covering the entire splenic parenchyma, while isolated tumors were less common. The pathological manifestations of SA vary, and there are differences in the same case and among different cases (20). Based on the analysis of previous literature, SA is mainly classified into four types as follows: 1) SA presents as atypical vascular endothelial cells, arranged in a spongy or honeycomb-like pattern (21); 2) Tumor cells are arranged in a porous pattern; 3) Malignant endothelial cells proliferate to form papillary lobes that extend into the vascular space; 4) The proliferation of endothelial cells in patches and the disordered arrangement of polygonal tumor cells form tumors. The stromal component of the tumor exhibits focal or diffuse cavernous hemangioma-like changes, lined by atypical endothelial cells demonstrating solid sarcomatoid and epithelioid growth patterns. The solid component resembles the appearance of fibrosarcoma or malignant fibrous histiocytoma. Immunological tests are usually helpful in confirming histological diagnosis. The common immunohistochemical manifestations of SA are positive for at least two endothelial markers (CD34, FVIIIRAg, VEGFR3 or CD31) and one histiocytic marker (CD68 or lysozyme) (22). The expression of these markers confirmed the endothelial phenotype of this malignant vascular tumor. The immunohistochemical test of this patient showed that both CD34 and CD31 related to vascular differentiation were positive, which was consistent with the reported results.

Splenectomy is the treatment of choice for SA. Regardless of whether SA patients have aggressive dissemination and metastasis or have already experienced splenic rupture, splenectomy can prolong the survival period of patients. In addition to surgery, radiotherapy, chemotherapy, and targeted immunotherapy are also important treatment methods (9). Studies have shown that the survival outcomes SA vary significantly depending on the treatment modality: 1) Surgery alone: The median overall survival (OS) of patients is 3.7–7 months, and whether splenic rupture occurs before surgery is an independent risk factor (5). This is because patients not only face the risk of immediate death due to hemorrhagic shock and disseminated intravascular coagulation, but splenic tumor rupture also promotes peritoneal dissemination and hematogenous metastasis of the tumor; 2) Surgery + chemotherapy: The European Society for Medical Oncology (ESMO) guidelines for soft tissue and visceral sarcoma recommend the use of paclitaxel/gemcitabine for the treatment of angiosarcoma. In addition, the combination of paclitaxel/gemcitabine and docetaxel can also be used as a treatment for angiosarcoma. According to the clinical practice guidelines for soft tissue sarcoma of the Japanese Orthopaedic Association, the combined application of doxorubicin and taxanes is also effective in the treatment of angiosarcoma. According to a study by Li et al. (5), after using a chemotherapy regimen postoperatively, the median overall survival is extended to 12–14 months; 3) Surgery + targeted immunotherapy: receptor tyrosine kinase inhibitors can target and bind to vascular endothelial growth factor (VEGF) receptors, thereby inhibiting tumor angiogenesis and tumor cell proliferation. PD-1 inhibitors can bind to PD-1 receptors and block their interaction with PD-L1 and PD-L2, thus releasing the suppression of immune responses mediated by the PD-1 pathway, including anti-tumor immune responses, and enabling the immune system to better attack and kill tumor cells. The NCCN Clinical Practice Guidelines in Oncology: Soft Tissue Sarcoma recommends the combination of anti-angiogenic drugs and PD-1 inhibitors for advanced unresectable or metastatic soft tissue sarcomas (especially subtypes such as angiosarcoma and synovial sarcoma) (23), stating that this combination can improve efficacy and has better safety than traditional chemotherapy. Xu et al. (6)reported that 1 patient with metastasis achieved CR after six cycles of targeted immunotherapy. This case confirms that immune checkpoint inhibition and anti-angiogenesis have a synergistic effect. Meanwhile, the Ki-67 index of this patient is approximately 30%, suggesting a high tumor proliferation activity. Therefore, this patient received the targeted immunotherapy regimen of anlotinib + toripalimab. Regrettably, the patient developed lumbosacral pain after receiving three cycles of treatment, and bone metastasis was considered based on CT examination. Subsequently, the patient was discharged from our hospital and went to another hospital for treatment, so we were unable to continue the follow - up. This is a shortcoming of this article.

## Conclusion

SA, as an extremely rare malignant tumor, is prone to misdiagnosis and missed diagnosis due to its lack of specific clinical manifestations, and SA has a very poor prognosis. The average survival period of patients is less than 6 months. The diagnosis of SA mainly relies on pathological examination and immunohistochemical analysis. Meanwhile, the clinical manifestations and imaging features of patients need to be comprehensively considered for multi-dimensional assessment. At present, the main approach to improving the survival rate of patients is to perform splenectomy early, supplemented by targeted immunotherapy. However, we still need to explore more effective treatment strategies to increase the survival time of patients in the future.

## Data Availability

The original contributions presented in the study are included in the article/supplementary material. Further inquiries can be directed to the corresponding author.
